# 

**Published:** 1874-08

**Authors:** 


### CONTENTS:

ART. I. To prevent Rotation in Fracture of the Femur, etc. By J. E. O'Brien, M.D. .... 449
ART. II. Another Case of Habitual Regurgitation. By G. W. Emery, M.D. 450
ART. III. Case of Ascites, treated by Introduction of Drainage Tube. By J. M. West, M.D. .... 451
ART. IV. Report on Surgery. By J. L. Hays, M.D. .... 453
ART. V. Case of Uterine Hemorrhage. By L. J. Willien, M.D. .... 457
ART. VI. A Case of Perforation of Bladder. By L. J. Willien, M.D. ... 460
ART. VII. Food for Infants. A Review of the Review. By A. S. von Mansfelde, M.D. .... 462
ART. VIII. New Treatment of Burns. By M. O. Baldwin, M.D. .... 469
SELECTIONS .... 470
THE HOSPITALS .... 473
REPORTS OF SOCIETIES .... 481
EDITORS' BOOK TABLE .... 501
EDITORIAL .... 506
MORTALITY STATISTICS .... 510

